# A Data-Driven Unsupervised Framework for Discovering Interpretable Gaze-Based Behavioral Pseudo-Zones in Children with Autism Spectrum Disorder

**DOI:** 10.3390/diagnostics16142176

**Published:** 2026-07-13

**Authors:** Rahaf Alrowithi, Haneen Banjar, Nofe Alganmi

**Affiliations:** 1 Computer Science Department, Faculty of Computing and Information Technology, King Abdulaziz University, Jeddah 21589, Saudi Arabia; hrbanjar@kau.edu.sa (H.B.); nalghanimi@kau.edu.sa (N.A.); 2 Center of Research Excellence in Artificial Intelligence and Data Science, King Abdulaziz University, Jeddah 21589, Saudi Arabia; 3 Institute of Genomic Medicine Sciences, King Abdulaziz University, Jeddah 21589, Saudi Arabia; 4 Centre of Artificial Intelligence in Precision Medicines, King Abdulaziz University, Jeddah 21589, Saudi Arabia

**Keywords:** autism spectrum disorder, eye-tracking, unsupervised learning, behavioral state discovery, interpretable artificial intelligence, gaze behavior, clustering, behavioral patterns

## Abstract

**Background/Objectives**: Children with autism spectrum disorder (ASD) often exhibit differences in attention regulation and visual behavior. However, many ASD eye-tracking datasets lack reliable moment-to-moment behavioral or emotional annotations, limiting the direct application of supervised learning approaches. To address this challenge, this study proposes an interpretable gaze-based unsupervised framework for discovering behavioral pseudo-zones from unlabeled ASD eye-tracking data. **Methods**: Raw gaze recordings from ASD participants were segmented into fixed temporal windows and represented using interpretable gaze features, including gaze dispersion, fixation duration, tracking quality, motion ratio, pupil size, and gaze velocity measures. Multiple clustering models and alternative temporal window sizes were systematically compared, including K-means, Gaussian Mixture Modeling (GMM), Agglomerative Clustering, and HDBSCAN. **Results**: Among the evaluated configurations, the combination of 1000 ms windows with K-means clustering (k = 4) was retained as the final exploratory configuration. Although alternative solutions achieved slightly stronger internal validation metrics, the selected configuration provided a more interpretable four-zone structure while maintaining acceptable clustering quality. The final retained solution produced four interpretable behavioral pseudo-zones with statistically significant differences across all extracted gaze features according to the Kruskal–Wallis test (*p* < 0.05). A PCA projection further supported the exploratory structure of the discovered pseudo-zones, with the first two principal components explaining 72.3% of the total variance. **Conclusions**: The findings demonstrate that unlabeled ASD gaze data can be organized into interpretable behavioral pseudo-zones using an unsupervised and transparent feature-based framework. This work contributes a data-driven and interpretable framework for future gaze-based behavioral analysis and autism-related AI research.

## 1. Introduction

Autism spectrum disorder (ASD) is associated with differences in social communication, sensory processing, and patterns of attention. Many autistic children also face challenges in emotional regulation during learning and everyday interaction. These challenges motivate the development of non-invasive computational approaches that can help characterize behavioral patterns and potentially support future assistive applications.

Eye tracking is a promising modality for this purpose because it captures where a child looks, how long gaze remains stable, how widely gaze is distributed across a scene, and how much visual motion occurs over time. Prior research has shown that gaze behavior in ASD is often atypical and may be closely related to emotion perception, attention, and social information processing [1,2]. In addition, previous work using the same broader eye-tracking research context has shown that gaze-based data can be useful for visual attention modeling and ASD-related computational analysis [3].

However, a common limitation in ASD eye-tracking research is the absence of reliable moment-to-moment emotional labels. As a result, building supervised emotion-recognition models directly from raw gaze data is often impractical. Existing work in autism-related affective computing has largely relied on explicit annotations, multimodal inputs, or supervised learning settings [4,5]. This leaves an important gap for ASD gaze datasets in which only unlabeled or weakly labeled eye-tracking signals are available.

To address this gap, the current study adopts a gaze-based unsupervised approach. Instead of predicting predefined emotional labels, the goal is to discover interpretable latent behavioral patterns directly from unlabeled gaze windows. These patterns are treated as exploratory pseudo-zones derived from gaze behavior rather than as clinically verified emotional diagnoses.

To facilitate interpretation, the final discovered clusters were mapped post hoc to four color-based pseudo-zones (Blue, Green, Yellow, and Red). This mapping was inspired by the Zones of Regulation framework and previous autism-related work using similar color-based emotional regulation concepts [4]. Importantly, these zone names were not used as ground-truth labels during model development. Instead, they served only as an interpretive framework for describing the behavioral characteristics of the discovered clusters.

Therefore, the aim of this study is to construct a structured gaze-window dataset from ASD eye-tracking recordings and apply an unsupervised framework to discover four interpretable behavioral pseudo-zones. More specifically, the study segments raw gaze data into fixed 1000 ms windows, extracts gaze-based behavioral features from each window, compares multiple clustering models and alternative temporal window lengths, and retains a four-cluster K-means solution as the final exploratory configuration based on clustering quality, interpretability, and consistency with the adopted pseudo-zone framework. The final clustering solution is then interpreted using the Blue/Green/Yellow/Red pseudo-zone framework and evaluated through internal validation, relative comparison across clustering models, comparison across alternative window lengths, interpretability analysis of cluster profiles, and statistical comparison across the final clusters. The retained solution was further examined through statistical testing and low-dimensional visualization using Principal Component Analysis (PCA).

In this way, the study contributes a data-driven framework for discovering latent behavioral patterns from unlabeled ASD gaze data. Rather than proposing a direct clinical emotion recognition system, the work is positioned as an exploratory and interpretable behavioral modeling stage that may support future gaze-based behavioral analysis, hypothesis generation, and emotion-aware assistive research in ASD.

## 2. Related Work

Previous research has shown that affective computing systems developed for the general population often do not transfer well to children with autism spectrum disorder (ASD), partly because emotional and behavioral expressions in ASD may differ from neurotypical patterns [4,5]. This has motivated ASD-specific studies in emotion detection, gaze analysis, and behavioral assessment.

A key related study is CALMED, a multimodal annotated dataset for emotion detection in children with autism [4]. CALMED is especially relevant to the present work because it adopts a color-based emotional regulation framework consisting of Blue, Green, Yellow, and Red zones. This framework inspired the post-hoc interpretation strategy used in the present study, although no emotional labels from CALMED were transferred to the current dataset.

Other work has directly examined the relationship between eye gaze and emotion perception in ASD. For example, Wang et al. showed that gaze patterns in children with ASD vary according to the sequence and intensity of emotional presentation, supporting the idea that visual gaze carries meaningful information about emotion perception processes [1]. Similarly, Van der Donck et al. investigated automatic emotion processing in children with autism using eye tracking and found that gaze behavior can reveal subtle differences in visual exploration strategies during facial emotion recognition [2].

From a broader eye-tracking perspective, Kong et al. reported that children with ASD and typically developing children exhibit different fixation patterns across several visual conditions, reinforcing the value of gaze-based features in data-driven behavioral analysis [6]. In addition, the Eye-Tracking Dataset to Support the Research on Autism Spectrum Disorder provided a publicly available eye-tracking dataset for ASD-related research [3]. The present study utilizes this publicly available dataset and transforms the raw gaze recordings into a structured window-level behavioral dataset through temporal segmentation and feature extraction. This enables the application of unsupervised clustering methods for latent behavioral pattern discovery. The dataset includes detailed gaze records and eye-movement measures collected during eye-tracking experiments, supporting further investigation of gaze behavior and computational analysis in autism research. These studies support the relevance of using gaze-derived summaries as computational features for identifying meaningful latent behavioral structures in ASD-related data.

Further evidence also shows that eye-tracking data can support machine learning-based differentiation between ASD and typical development in structured interaction settings [7] and that gaze-based measures may relate to clinically meaningful behavioral characteristics in children with ASD [8,9]. At the same time, methodological studies have emphasized that eye-tracking results in autism research can be affected by data quality and measurement conditions, reinforcing the need for careful preprocessing and cautious interpretation [10].

Overall, prior literature has mainly focused on supervised emotion recognition, multimodal annotated datasets, ASD-versus-TD classification, or descriptive comparisons of gaze behavior in ASD. In contrast, the present study investigates whether interpretable behavioral pseudo-zones can be discovered directly from unlabeled ASD gaze recordings using an unsupervised framework. The study further evaluates alternative temporal window lengths and multiple clustering approaches before retaining a final four-cluster K-means configuration for exploratory behavioral interpretation. Thus, the contribution of this work is not a direct emotion classifier, but a transparent and data-driven framework for discovering gaze-based behavioral pseudo-zones that may support future ASD behavioral analysis and assistive research.

## 3. Materials and Methods

**Figure 1:**
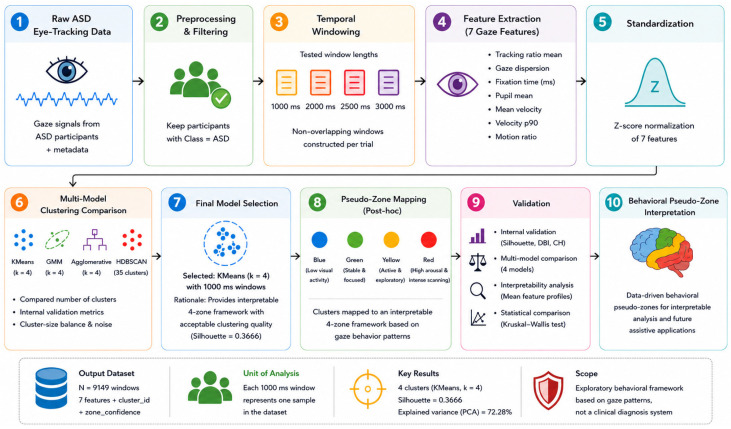
Workflow of the exploratory gaze-based unsupervised framework, from raw ASD eye-tracking data to behavioral pseudo-zone interpretation and validation.

The present study was designed as an exploratory preprocessing and data-driven representation stage for future emotion-aware systems, rather than as a direct solution for emotion regulation or a clinically validated emotion recognition system. The goal of this stage was to transform raw ASD eye-tracking recordings into a structured and interpretable gaze-window representation that could support later behavioral analysis. The overall workflow of the implemented framework is summarized in Figure 1.

### 3.1. Data Source and Participant Selection

This study used the public Eye Tracking Autism dataset, which contains raw eye-tracking recordings and participant-level metadata collected to support autism research. The dataset includes 59 children in total, comprising 29 children diagnosed with autism spectrum disorder (ASD) and 30 typically developing (TD) children. Participant metadata include a unique participant identifier, gender, age, diagnostic class, and Childhood Autism Rating Scale (CARS) score [3].

For the present study, only participants labeled as ASD were retained because the objective was to explore gaze-based behavioral patterns within ASD rather than to compare ASD and TD groups. Within the ASD subset, 25 participants were male, and 4 were female. Participant ages ranged from 2.7 to 12.3 years, and CARS scores ranged from 17 to 45 [3].

According to the dataset documentation and the associated publication, eye-movement recordings were collected using an SMI Red-M eye tracker operating at a sampling rate of 60 Hz. Participants were seated approximately 60 cm from a 17-inch monitor with a screen resolution of 1280 × 1024 pixels. A five-point calibration procedure was performed before data collection. The experimental protocol included multiple visual stimuli, including photographs, videos, static scenes, dynamic scenes, faces, objects, cartoons, and joint-attention stimuli. The dataset is distributed across 25 raw eye-tracking CSV files together with a separate participant metadata file [3].

Participant identifiers from the metadata file were standardized and matched with the raw eye-tracking recordings. Although the metadata contained 29 ASD participants, valid raw gaze recordings were successfully linked for 27 ASD participants and were therefore included in the final analysis. Participants 12 and 16 were not represented in the linked raw gaze records available to the preprocessing pipeline. After filtering to ASD participants, the raw dataset contained 34 unique trial labels distributed across the available recording files.

The preprocessing pipeline began by reading the raw eye-tracking files and participant metadata. Variables were selected based on their direct relevance to temporal segmentation, gaze-position estimation, eye-movement characterization, pupil-based measurements, and participant-level tracking quality. The retained variables included recording time, participant identifier, trial label, stimulus identifier, tracking ratio, eye-movement event category, pupil diameter measurements, and left- and right-eye point-of-regard coordinates. Variables not directly related to gaze-feature extraction were excluded from the analysis.

Samples with missing gaze coordinates for both eyes were removed. For each sample, binocular gaze coordinates were calculated by averaging the available left- and right-eye gaze positions. Mean pupil diameter was calculated as the average of the left- and right-eye pupil measurements. Non-positive pupil diameter values were treated as invalid measurements and converted to missing values prior to feature extraction. This approach follows established eye-tracking preprocessing practices for combining binocular gaze information into a single gaze estimate [11].

Relative recording time was computed separately within each participant–source file–trial combination and segmented into non-overlapping temporal windows of 1000 ms. For each window, seven gaze-based behavioral features were extracted: tracking_ratio_mean, gaze_dispersion, fixation_time_ms, pupil_mean, mean_vel, vel_p90, and motion_ratio. Windows containing no meaningful gaze signal or missing values in the extracted features were excluded. Participant-level metadata, including gender, age, and CARS score, were then merged with the window-level feature table.

The resulting analytical dataset contained 9149 valid gaze windows. The retained dataset included participant descriptors, temporal-window variables, and seven extracted gaze-based behavioral features. Following clustering and post-hoc interpretation, three additional variables (cluster_id, zone, and zone_confidence) were generated, resulting in a final dataset containing 21 columns.

Data preprocessing, feature extraction, clustering, statistical analysis, and visualization were performed using Python 3.12.13 with pandas 2.2.2, NumPy 2.0.2, SciPy 1.16.3, scikit-learn 1.6.1, and Matplotlib 3.10.0.

### 3.2. Preprocessing and Temporal Windowing

For each Participant, source_file, and Trial combination, samples were sorted chronologically. A relative time variable, t_rel, was computed by subtracting the minimum recording time within each recording segment. The raw eye-tracking signal was then segmented into fixed, non-overlapping temporal windows according to

$$window_{index}=floor(\frac{t_{rel}}{WINDOW_{MS}})$$

For each temporal window, the following structural variables were computed: window_start_ms, window_end_ms, window_time_ms, and samples. Non-overlapping windows were adopted to preserve a clear temporal partitioning of the gaze signal and to avoid duplicate observations across adjacent windows.

To examine the influence of temporal granularity on the resulting clustering structure, multiple window lengths were evaluated, including 1000 ms, 2000 ms, 2500 ms, and 3000 ms. Each window configuration was processed using the same preprocessing, feature extraction, and clustering pipeline. Comparative evaluation was performed using internal clustering validity metrics and cluster interpretability criteria.

Based on this comparison, the 1000 ms configuration was retained for the final framework. Although alternative configurations achieved slightly stronger values for some internal validation metrics, the 1000 ms setting preserved higher temporal granularity and supported the exploratory four-zone behavioral interpretation adopted in this study.

### 3.3. Gaze Aggregation and Feature Extraction

Samples with missing gaze coordinates for both eyes were removed prior to feature extraction. For each remaining sample, a unified binocular gaze point was computed by averaging the available left- and right-eye point-of-regard coordinates, resulting in the variables Gaze_X and Gaze_Y. Similarly, a unified pupil measurement was computed as the average of the left- and right-eye pupil diameters. This aggregation strategy was adopted because binocular differences were not the focus of the present study, and the objective was to derive a stable gaze representation for downstream behavioral analysis [12].

Following temporal segmentation, each window was summarized using seven gaze-based behavioral features: tracking_ratio_mean, gaze_dispersion, fixation_time_ms, pupil_mean, mean_vel, vel_p90, and motion_ratio. These features were selected because they capture complementary aspects of gaze behavior, including tracking quality, spatial gaze variability, fixation behavior, pupil dynamics, and eye-movement activity. Detailed mathematical definitions and computation procedures for all extracted features are provided in Section 3.4.

In addition to the extracted behavioral features, the dataset retained participant-level variables (Participant, Gender, Age, and CARS_Score), trial identifiers, and temporal-window variables (window_index, window_start_ms, window_end_ms, window_time_ms, and samples). As a result, the analytical dataset contained 18 columns prior to clustering. After clustering and post-hoc interpretation, three additional variables (cluster_id, zone, and zone_confidence) were added, resulting in a final dataset containing 21 columns.

### 3.4. Core Clustering Features

tracking_ratio_mean was computed as the mean value of the eye-tracking quality measure (Tracking Ratio [%]) within each temporal window. This feature reflects the proportion of valid gaze samples successfully captured by the eye-tracking system.

gaze_dispersion was computed from the binocular gaze coordinates as

$$gaze\_dispersion=\sqrt{Var\left(Gaze\_X\right)+Var\left(Gaze\_Y\right)}$$

where Gaze_X and Gaze_Y represent the aggregated binocular gaze coordinates within the window.

Larger values indicate a wider spatial distribution of gaze across the visual stimulus. fixation_time_ms was estimated from the proportion of samples classified as fixation in either the left-eye or right-eye event annotations:

fixation_time_ms=fixation_proportion×window_time_ms

This feature provides an approximate measure of the amount of time spent in fixation behavior within the window.

pupil_mean was computed as the average binocular pupil diameter across all samples within the window.

Eye-movement velocity was estimated from frame-to-frame gaze displacement divided by elapsed recording time:

$$vel=\frac{\sqrt{{dx}^{2}+{dy}^{2}}}{dt}$$

where dx and dy represent consecutive changes in gaze position, and dt represents the elapsed recording time between consecutive samples.

Based on these velocity values, mean_vel was computed as the mean velocity within the window, whereas vel_p90 represented the 90th percentile of velocity values and was used to characterize brief high-velocity gaze movements.

motion_ratio was computed as

$${motion}_{ratio}=\frac{Number\;of\;samples\;with\;vel>0}{Total\;number\;of\;samples}$$

This feature quantifies the proportion of the window spent in gaze motion rather than near-static viewing behavior.

Together, these features summarize complementary aspects of gaze behavior, including tracking quality, spatial gaze variability, fixation behavior, pupil dynamics, and short-term gaze motion. These features were selected because they provide interpretable behavioral descriptors that can be derived directly from the raw eye-tracking recordings.

To reduce over-interpretation of gaze-derived patterns, the discovered pseudo-zones were operationally interpreted using observable behavioral gaze indicators rather than direct emotional assumptions. For example, higher fixation duration together with lower gaze dispersion was interpreted as organized visual focus behavior, whereas frequent gaze shifts and elevated dispersion were interpreted as visually scattered attentional behavior. These operational interpretations were intended as exploratory behavioral descriptions rather than clinically validated emotional states.

### 3.5. Standardization and Clustering Models

Before clustering, the seven behavioral gaze features were converted to numeric form and standardized using z-score normalization. This step ensured that all features contributed comparably to distance-based clustering and prevented variables with larger numerical ranges from disproportionately influencing the clustering process.

To reduce dependence on a single clustering assumption, four clustering models were evaluated: K-means, Gaussian Mixture Modeling (GMM), Agglomerative Clustering, and HDBSCAN [11,13]. The comparison considered the number of clusters produced, the presence of noise points, internal clustering validity metrics, and cluster-size balance.

Additional analyses were performed using K-means with K values ranging from 2 to 6. Internal clustering validity was assessed using the Silhouette Score, Davies–Bouldin Index, and Calinski–Harabasz Index. Although alternative K values achieved slightly stronger values for some internal validation metrics, the four-cluster solution was retained because it enabled a more detailed behavioral characterization and supported the exploratory pseudo-zone framework while maintaining acceptable clustering quality. Quantitative comparisons across K values are reported in Section 4.

K-means was selected as the final clustering model because it produced a stable four-cluster solution without noise points and provided the most suitable balance between clustering validity, cluster interpretability, and consistency with the study objectives. The alternative clustering models were retained as comparative baselines for evaluating the robustness of the clustering structure.

Although the selected model produced four clusters, these clusters should not be interpreted as directly observed emotional categories. Instead, they represent data-driven behavioral groupings derived from gaze features and subsequently interpreted within the adopted pseudo-zone framework.

After clustering, each temporal window received a numeric cluster_id. Cluster centers were then compared using the mean values of mean_vel, vel_p90, motion_ratio, and gaze_dispersion. These feature profiles were used to support the post-hoc interpretation of the discovered clusters and their mapping to the four exploratory pseudo-zones.

### 3.6. Model Validation

Because this study used unsupervised learning, no ground-truth emotional labels were available for direct accuracy-based evaluation. Therefore, the selected clustering solution was evaluated using complementary strategies: internal validation, relative model comparison, interpretability validation, and statistical comparison across the final clusters. In the present exploratory study, validation was treated as part of the methodology because it directly informed the assessment of the selected clustering solution.

#### 3.6.1. Internal Validation

Internal validation examined the structural quality of the clustering solutions using the Silhouette Score, Davies–Bouldin Index, and Calinski–Harabasz Score. These metrics were used to compare clustering quality across models and across alternative window lengths.

#### 3.6.2. Relative Model Comparison

To assess whether the discovered cluster structure was strongly dependent on a single clustering algorithm, K-means was compared with Gaussian Mixture Modeling (GMM), Agglomerative Clustering, and HDBSCAN. This comparison was used to evaluate whether the retained four-cluster solution remained reasonable under alternative clustering assumptions.

#### 3.6.3. Interpretability Validation Using Mean Feature Profiles

Interpretability validation focused on whether the discovered clusters could be meaningfully described through their feature profiles. This was particularly important because the purpose of the study was not only to cluster the data mathematically but also to derive meaningful gaze-based behavioral pseudo-zones.

For the final retained solution, cluster-level mean feature profiles were examined across the seven behavioral gaze features. Differences in tracking quality, fixation behavior, gaze dispersion, pupil dynamics, and gaze motion were used to assess whether the discovered clusters exhibited distinct and interpretable behavioral patterns. The detailed feature profiles and their pseudo-zone interpretations are reported in Section 4.

#### 3.6.4. Exploratory Visualization Using PCA

To provide a low-dimensional visualization of the discovered pseudo-zones, Principal Component Analysis (PCA) was applied to the standardized feature matrix. The first two principal components were used to project the windows into a two-dimensional space for visual inspection of cluster separation and overlap. PCA was used only as an exploratory visualization tool and was not involved in the clustering process itself.

#### 3.6.5. Statistical Analysis of Cluster Differences

To further examine whether the final discovered clusters differed meaningfully across the behavioral gaze features, a statistical comparison was performed using the Kruskal–Wallis test. This non-parametric test was selected because the final solution involved more than two clusters and the feature distributions were not assumed to follow normality. The test was applied to the seven main behavioral gaze features in the final retained solution.

### 3.7. Zone Confidence

A zone_confidence score was computed from the Euclidean distance between each window and the centroid of its assigned K-means cluster in the standardized feature space. Let (d_i) denote the distance between window (i) and its assigned cluster centroid.

Distances were normalized using min–max normalization:

$$d_{i}^{norm}=\frac{d_{i}-d_{min}}{d_{max}-d_{min}}$$

The final confidence score was then computed as

$$Zone\_confidence=1-d_{i}^{norm}$$

where d_min and d_max represent the minimum and maximum assigned-centroid distances observed across all windows. Consequently, windows located closer to their cluster centroid received confidence values closer to 1, whereas windows located farther from the centroid received lower confidence values closer to 0.

The zone_confidence score was introduced as an exploratory measure of cluster-membership strength and was used to quantify how closely each window matched the characteristic feature profile of its assigned pseudo-zone.

## 4. Results

This section presents the structure of the final processed dataset, the distribution of the discovered pseudo-zones, the main behavioral differences observed across the resulting clusters, and the statistical comparison across the final retained solution.

### 4.1. Final Dataset Structure

Under the final selected configuration, the processed dataset contained 9149 gaze windows derived from 27 ASD participants. Prior to clustering, the analytical dataset contained 18 columns, including participant descriptors, temporal-window variables, and seven extracted gaze-based behavioral features. Following clustering and post hoc interpretation, three additional variables (cluster_id, zone, and zone_confidence) were generated, resulting in a final dataset containing 21 columns.

The final retained temporal setting used throughout the main analysis was 1000 ms. The following analyses examine the clustering structure obtained from this final dataset.

### 4.2. Comparison of Clustering Models

Before comparing clustering models, alternative temporal window lengths were evaluated to examine the influence of temporal granularity on the resulting clustering structure. As summarized in Table 1, the 1000 ms configuration achieved the strongest overall clustering performance among the evaluated window lengths. Specifically, the 1000 ms setting produced the highest Silhouette score (0.3666) and the lowest Davies–Bouldin Index (1.2020), indicating better cluster separation and compactness than the alternative temporal resolutions. Therefore, the 1000 ms window length was retained for all subsequent analyses.

Four clustering models were then compared under the retained 1000 ms window setting: K-means, Gaussian Mixture Modeling (GMM), Agglomerative Clustering, and HDBSCAN. The comparison considered the number of clusters produced, the number of noise points, internal validity metrics, and cluster-size balance.

As summarized in Table 2, HDBSCAN achieved the highest Silhouette score and lowest Davies–Bouldin Index; however, it generated 35 clusters together with 3351 noise points, resulting in a highly fragmented clustering structure that was less compatible with the adopted four-zone exploratory framework. GMM produced four clusters but showed the weakest overall clustering validity among the evaluated alternatives. Agglomerative Clustering generated four clusters with performance comparable to K-means, although K-means achieved slightly better overall clustering validity and cluster balance.

K-means provided the most suitable balance between cluster interpretability, cluster stability, absence of noise points, and compatibility with the adopted four-zone exploratory framework. Therefore, K-means was retained as the final clustering model for the remainder of the study.

### 4.3. Comparison Across Different Values of K

To further examine the effect of the number of clusters on the resulting clustering structure, K-means solutions with K values ranging from 2 to 6 were evaluated. The comparison considered internal clustering validity metrics, including the Silhouette Score, Davies–Bouldin Index (DBI), Calinski–Harabasz Score (CHS), and cluster-size distribution.

As summarized in Table 3, the K = 3 solution achieved the highest Silhouette Score (0.4054) and the highest Calinski–Harabasz Score (5576.25), indicating the strongest overall internal clustering validity among the evaluated alternatives. The K = 2 solution produced the lowest Davies–Bouldin Index (0.9664), while larger values of K generally resulted in reduced clustering quality.

Despite the stronger internal validity metrics observed for K = 3, the K = 4 solution was retained for the final exploratory framework. The four-cluster structure provided a more detailed yet interpretable behavioral partition of the gaze data while remaining compatible with the adopted four-zone interpretive framework (Blue, Green, Yellow, and Red). In addition, the retained K = 4 solution maintained acceptable clustering validity and produced clusters of sufficient size for subsequent interpretation.

The retained K = 4 solution therefore represented a practical compromise between clustering validity, behavioral interpretability, and compatibility with the adopted exploratory pseudo-zone framework.

### 4.4. Pseudo-Zone Distribution

**Figure 2:**
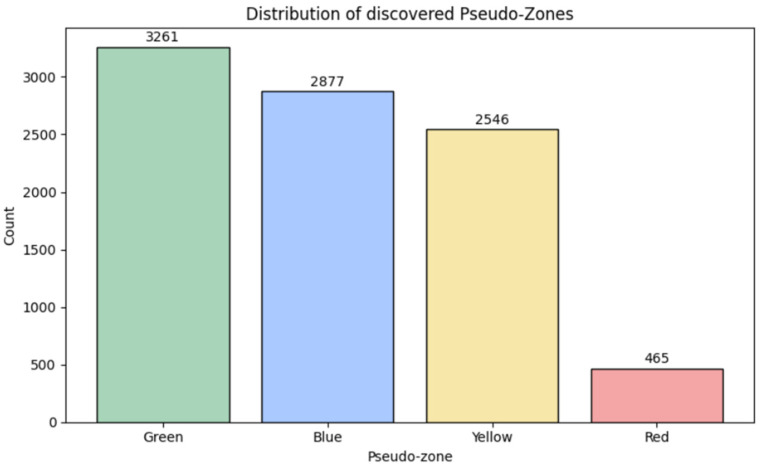
Distribution of the discovered pseudo-zones across the final processed gaze-window dataset.

The final retained K-means solution produced four behavioral pseudo-zones. As summarized in Table 4 and Figure 2, the Green pseudo-zone represented the largest proportion of windows (35.64%), followed by the Blue pseudo-zone (31.45%), the Yellow pseudo-zone (27.83%), and the Red pseudo-zone (5.08%).

Overall, the distribution suggests that the majority of gaze windows were assigned to Green and Blue patterns, while Yellow and Red represented progressively less frequent behavioral patterns. Importantly, all four pseudo-zones were represented by a sufficient number of windows to support subsequent behavioral interpretation and statistical comparison.

The observed distribution indicates that the discovered clustering structure was not dominated by a single cluster and produced a reasonably balanced partition of the gaze-window dataset. This balance supports the interpretability of the retained four-cluster solution within the adopted exploratory pseudo-zone framework.

### 4.5. Behavioral Interpretation of the Discovered Clusters

The behavioral feature profiles of the discovered pseudo-zones are summarized in Table 5 as mean ± SD values. Clear behavioral differences were observed across the four groups, supporting the interpretability of the retained four-cluster solution.

The Green pseudo-zone exhibited the highest tracking ratio mean (77.86), the longest fixation time (828.06 ms), and a high motion ratio (0.98). In addition, it showed relatively low gaze velocity and moderate gaze dispersion. Collectively, these characteristics suggest a stable and sustained visual attention pattern.

The Blue pseudo-zone was characterized by the lowest tracking ratio mean (27.45), minimal fixation time (8.74 ms), extremely low motion ratio (0.03), and the lowest gaze velocity measures. This profile reflects a low-motion and low-engagement gaze pattern with limited visual activity.

The Yellow pseudo-zone showed intermediate tracking quality together with increased gaze dispersion (330.50), moderate fixation duration (445.39 ms), and elevated gaze velocity measures. Compared with the Green pseudo-zone, the Yellow pseudo-zone demonstrated a more exploratory and dynamic visual scanning pattern.

The Red pseudo-zone exhibited the highest gaze dispersion (432.13), the highest mean velocity (11.23), and the highest velocity p90 (41.75). It also showed shorter fixation durations than both the Green and Yellow pseudo-zones. These characteristics suggest a highly dynamic and spatially dispersed gaze pattern relative to the other discovered groups.

Overall, the four pseudo-zones represent distinct behavioral gaze profiles ranging from stable and sustained visual attention (Green) to low-activity gaze behavior (Blue) to increasingly dynamic visual exploration patterns (Yellow and Red). Importantly, these interpretations are based on gaze-derived behavioral characteristics and should be understood as exploratory behavioral pseudo-zones rather than clinically validated emotional states.

### 4.6. Visual Comparison of Selected Behavioral Features

**Figure 3:**
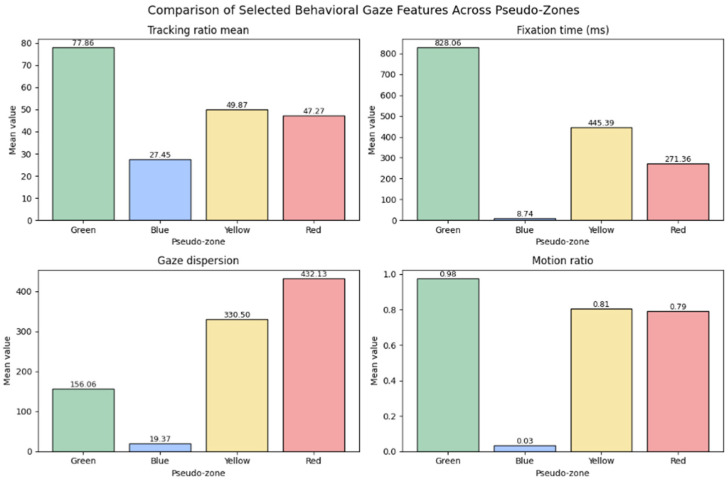
Comparison of selected behavioral gaze features across the discovered pseudo-zones.

Figure 3 presents a comparison of selected behavioral gaze features across the discovered pseudo-zones. Consistent with the numerical summaries reported in Table 5, the Green pseudo-zone exhibited the highest tracking ratio and fixation duration, together with a high motion ratio. The Blue pseudo-zone showed the lowest values across most behavioral measures, particularly tracking ratio, fixation duration, and motion ratio. The Yellow pseudo-zone demonstrated increased gaze dispersion and intermediate gaze dynamics, whereas the Red pseudo-zone exhibited the highest gaze dispersion and velocity-related measures among the discovered groups.

**Figure 4:**
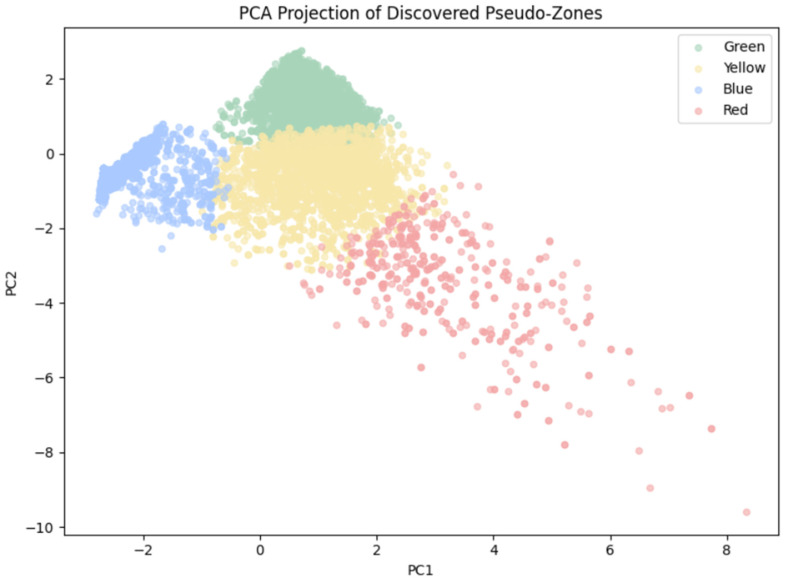
PCA projection of the discovered pseudo-zones based on the seven behavioral gaze features.

To further examine the overall structure of the discovered pseudo-zones, Principal Component Analysis (PCA) was applied to the seven behavioral gaze features. The first two principal components explained 72.28% of the total variance in the dataset (PC1 = 43.74%, PC2 = 28.54%), as shown in Figure 4.

The PCA projection revealed partial separation among the discovered pseudo-zones. The Green and Blue pseudo-zones occupied relatively compact regions of the feature space, whereas the Yellow pseudo-zone showed greater overlap with the neighboring pseudo-zones. The Red pseudo-zone appeared more distinct within the principal component space, consistent with its elevated gaze dispersion and velocity measures reported in Table 5.

Overall, the visual analyses support the existence of meaningful behavioral differences among the discovered pseudo-zones. However, these groupings should be interpreted as exploratory gaze-derived behavioral patterns rather than clinically validated emotional categories.

### 4.7. Statistical Comparison Across the Final Clusters

To further assess whether the retained clusters differed significantly across the behavioral gaze features, a Kruskal–Wallis test was applied to the final 1000 ms + K-means solution. As shown in Table 6, the results indicated statistically significant differences across clusters for all seven main features: tracking_ratio_mean, gaze_dispersion, fixation_time_ms, pupil_mean, mean_vel, vel_p90, and motion_ratio (all *p* < 0.05). These findings provide additional evidence that the discovered clusters capture statistically distinct behavioral gaze patterns across all examined feature dimensions.

## 5. Discussion

The findings of this study suggest that meaningful latent structure can be discovered from unlabeled ASD eye-tracking data using an unsupervised framework. Although the dataset did not include clinically validated emotional labels, the final retained solution (1000 ms + K-means) produced four interpretable pseudo-zones that were supported by internal validation, comparison across alternative clustering models, comparison across alternative window lengths, and statistical differentiation across the main gaze features. This supports the idea that gaze-based clustering can provide a useful intermediate representation for analyzing latent behavioral patterns in ASD-related gaze data when direct supervision is unavailable.

The present findings are broadly consistent with prior work showing that gaze behavior in children with ASD contains meaningful information related to attention and emotion processing. For example, Wang et al. [1] showed that gaze patterns in children with ASD vary according to the sequence and intensity of emotional presentation, supporting the idea that visual gaze behavior is linked to emotion perception processes. Similarly, Van der Donck et al. [2] reported that eye-tracking measures can reveal subtle differences in visual exploration strategies during facial emotion processing in autism. In this sense, the current study extends prior findings by showing that even without explicit emotion labels, gaze-derived features can still be organized into interpretable behavioral groupings.

The study also complements CALMED [4], which used a multimodal annotated framework based on the Blue/Green/Yellow/Red zones of regulation. While CALMED relied on external annotation and multimodal input, the present work focused on unlabeled gaze-only data and used unsupervised learning to discover data-driven pseudo-zones. Therefore, the contribution of the current study is not to reproduce CALMED but to provide a different methodological perspective: how a zone-inspired interpretive framework can be applied after clustering, rather than being used as a predefined labeled target.

From a methodological perspective, the results support the value of transparent feature-based modeling in healthcare-related AI settings. The selected gaze features were interpretable and allowed the discovered pseudo-zones to be described in terms of tracking quality, fixation behavior, spatial dispersion, motion, and velocity. This is important because interpretability is especially valuable in exploratory AI systems intended for health-related applications, where black-box predictions may be harder to justify or trust. The comparison across clustering models further strengthened this choice: although some alternative models produced numerically strong values on selected metrics, K-means provided the best overall balance between four-cluster interpretability, acceptable internal validity, absence of noise points, and suitability for the adopted exploratory framework. Likewise, the comparison across alternative window lengths showed that the 1000 ms setting provided the strongest overall balance among the tested temporal configurations.

The final pseudo-zones were also statistically supported. Kruskal–Wallis testing showed significant differences across the retained clusters for all seven behavioral gaze features, indicating that the discovered groups were not only visually distinct in the descriptive tables and figures but also statistically differentiated. This supports the interpretation that the final clustering solution captured non-random structure in the gaze data rather than arbitrary partitioning.

The Green pseudo-zone showed the clearest organized profile, combining high tracking quality, long fixation time, and a strong motion ratio. By contrast, the Yellow pseudo-zone reflected a more scattered attentional pattern, while the Blue pseudo-zone reflected a low-tracking and low-motion gaze pattern. The Red pseudo-zone exhibited the highest gaze dispersion and velocity-related measures among the discovered groups, indicating a highly dynamic visual exploration pattern. Although it represented the smallest of the four pseudo-zones, it remained sufficiently represented in the dataset to support descriptive and statistical analysis. Nevertheless, as with all discovered groups in the present study, its interpretation should remain exploratory and grounded in gaze behavior rather than direct emotional inference.

Likewise, the Blue pseudo-zone should not be interpreted as a direct emotional state, since in this dataset it may also reflect periods of reduced tracking quality. These considerations reinforce the importance of treating the discovered zones as exploratory pseudo-zones rather than clinically validated emotional classes.

The broader contribution of this work lies in framing unsupervised gaze-based clustering as a data-driven representation layer for future ASD-support technologies. In contrast to direct emotion recognition systems, the current framework identifies structured behavioral patterns from raw gaze recordings without requiring frame-level emotion labels. This makes it potentially useful as a preprocessing or behavioral abstraction stage for future gaze-based behavioral analysis and assistive AI systems.

In addition, expert-informed behavioral feedback was considered during refinement of the pseudo-zone terminology and interpretation strategy in order to reduce potential over-interpretation of gaze-derived behavioral patterns.

Overall, the study contributes to the field by showing that unlabeled ASD gaze data can be transformed into structured and interpretable behavioral pseudo-zones using an unsupervised and transparent feature-based framework. Rather than assuming that clinically meaningful categories must be directly labeled in advance, the study shows that data-driven latent behavioral states can first be discovered from gaze structure and then interpreted cautiously within an external conceptual framework.

## 6. Limitations

This study has several limitations that should be considered when interpreting the findings.

### 6.1. Data Limitations

First, the discovered pseudo-zones were derived from unlabeled ASD eye-tracking data and were not based on clinically validated emotional ground truth. Therefore, the resulting zones should not be interpreted as confirmed emotional categories. In addition, the Blue pseudo-zone may combine genuinely low engagement with periods of reduced tracking quality, which makes its interpretation less specific. Although the Red pseudo-zone represented the smallest of the four discovered groups, its interpretation remains exploratory because the clustering process was based exclusively on gaze-derived behavioral features without external behavioral or emotional validation.

### 6.2. Methodological Limitations

Second, although the final retained framework selected the 1000 ms window setting after comparison across multiple alternative window lengths, the present study still relied on fixed windowing rather than adaptive segmentation. As a result, some longer-range or more complex temporal dynamics in gaze behavior may not have been fully captured. In addition, the selected feature set was designed to provide interpretable behavioral summaries, but it does not represent all possible gaze descriptors.

The study also compared multiple clustering models, including K-means, GMM, Agglomerative Clustering, and HDBSCAN, and included statistical comparison across the final clusters using the Kruskal–Wallis test. However, the final pseudo-zone mapping remained interpretive and post hoc rather than externally validated. Therefore, the resulting four-zone structure should still be understood as an exploratory behavioral framework rather than a definitive representation of the underlying behavioral structure.

An additional limitation is that multiple temporal windows were generated from the same participants across different trials. Therefore, the resulting windows cannot be considered fully independent observations. Although this study focused on exploratory clustering at the gaze-window level, future work may benefit from participant-level analyses or mixed-effects statistical frameworks that explicitly account for repeated observations from the same individuals.

### 6.3. Application Limitations

Third, the present work represents an exploratory preprocessing and representation stage rather than a deployed assistive or clinical system. The study did not include external ratings from therapists, caregivers, or the children themselves, and therefore the discovered pseudo-zones were not externally validated in real-world use settings. Accordingly, the proposed framework should be understood as a data-driven behavioral representation layer for future work, rather than as a direct emotion recognition tool, an emotion regulation support system, or a completed assistive technology implementation.

## 7. Conclusions

This study presented an exploratory gaze-based unsupervised framework for discovering interpretable behavioral pseudo-zones from unlabeled ASD eye-tracking data. By transforming continuous gaze recordings into fixed 1000 ms windows, extracting interpretable behavioral gaze features, comparing multiple clustering models, and retaining the most suitable final solution, the study produced a structured representation of gaze behavior that could be organized into four exploratory pseudo-zones interpreted using the Blue/Green/Yellow/Red framework.

The findings showed that unlabeled ASD gaze data can be grouped into interpretable latent behavioral patterns, even in the absence of frame-level emotional labels. The final retained solution (1000 ms + K-means) was supported by internal validation, comparison across alternative clustering models, comparison across alternative window lengths, interpretable feature-profile differences across the discovered pseudo-zones, and statistical comparison using the Kruskal–Wallis test. At the same time, these zones should be understood as exploratory behavioral representations rather than clinically validated emotional categories.

The main contribution of the study is the introduction of a transparent and data-driven behavioral representation layer for future ASD-related gaze-based behavioral analysis and affective computing research. Instead of directly predicting emotions, the proposed framework provides a structured intermediate stage that may support later gaze-based behavioral and emotion-aware analysis.

Future work should strengthen this framework through richer temporal gaze modeling, stronger external or expert-informed validation, and more comprehensive real-world evaluation. In addition, future studies may investigate how such gaze-based behavioral representations could be integrated into emotion-aware assistive systems while maintaining a careful distinction between exploratory behavioral inference and clinically validated emotional interpretation.

## Figures and Tables

**Table 1:** Comparison of clustering performance across temporal window sizes.

Window Size (ms)	Number of Windows	Silhouette Score	DBI	CHS
1000	9149	0.3666	1.2020	5118.98
2000	4965	0.3279	1.2606	2461.20
2500	4248	0.3017	1.3236	1842.18
3000	3203	0.3284	1.2137	1582.44

**Table 2:** Comparison of clustering models under the final 1000 ms window setting.

MODEL	CLUSTERS	Noise	Silhouette Score	Davies–Bouldin Index	Calinski–Harabasz Score	MIN SIZE	MAX SIZE
KMEANS	4	0	0.3666	1.2020	5118.98	465	3261
GMM	4	0	0.2315	1.7154	2972.77	526	3254
AGGLOMERATIVE	4	0	0.3499	1.2497	4519.02	334	3683
HDBSCAN	35	3351	0.4002	0.5843	327.43	30	4021

**Table 3:** Comparison of K-means clustering performance across different values of K.

K	Silhouette Score	DBI	CHS	Min Cluster Size	Max Cluster Size
2	0.3962	0.9664	5406.48	3005	6144
3	0.4054	1.1224	5576.25	1877	4272
4	0.3666	1.2020	5118.98	465	3261
5	0.3412	1.2237	4563.84	447	2857
6	0.2568	1.2707	4134.93	448	2327

**Table 4:** Distribution of the discovered pseudo-zones.

Pseudo-Zone	Count	Percentage (%)
Green	3261	35.64
Blue	2877	31.45
Yellow	2546	27.83
Red	465	5.08

**Table 5:** Mean ± SD behavioral gaze features across the discovered pseudo-zones.

Pseudo-Zone	Tracking Ratio (Mean ± SD)	Gaze Dispersion (Mean ± SD)	Fixation Time (ms) (Mean ± SD)	Motion Ratio (Mean ± SD)	Pupil Mean (Mean ± SD)	Mean Velocity (Mean ± SD)	Velocity p90 (Mean ± SD)
Green	77.86 ± 18.88	156.06 ± 103.05	828.06 ± 141.00	0.98 ± 0.07	3.83 ± 0.66	1.40 ± 0.94	2.80 ± 2.93
Blue	27.45 ± 19.93	19.37 ± 64.32	8.74 ± 47.61	0.03 ± 0.10	3.99 ± 0.65	0.18 ± 0.63	0.14 ± 0.89
Yellow	49.87 ± 21.86	330.50 ± 117.41	445.39 ± 231.02	0.81 ± 0.23	4.05 ± 0.63	4.06 ± 1.78	11.01 ± 7.10
Red	47.27 ± 22.95	432.13 ± 126.48	271.36 ± 253.94	0.79 ± 0.23	4.11 ± 0.69	11.23 ± 3.90	41.75 ± 14.99

**Table 6:** Kruskal–Wallis test results across the final K-means clusters.

Feature	Kruskal Statistic	*p*-Value	Significant at 0.05
TRACKING_RATIO_MEAN	4665.56	<0.001	Yes
GAZE_DISPERSION	6647.99	<0.001	Yes
FIXATION_TIME_MS	7459.20	<0.001	Yes
PUPIL_MEAN	227.96	<0.001	Yes
MEAN_VEL	7177.57	<0.001	Yes
VEL_P90	7288.12	<0.001	Yes
MOTION_RATIO	6921.59	<0.001	Yes

## Data Availability

The dataset analyzed in this study is publicly available on Kaggle under the “Eye Tracking Autism” dataset: https://www.kaggle.com/datasets/imtkaggleteam/eye-tracking-autism (accessed on 20 May 2026). The dataset originates from previously published research sources related to autism eye-tracking studies.

## Abbreviations

The following abbreviations are used in this manuscript:

ASD	Autism Spectrum Disorder
TD	Typically Developing
AI	Artificial Intelligence
HDBSCAN	Hierarchical Density-Based Spatial Clustering of Applications with Noise
GMM	Gaussian Mixture Model
DBI	Davies–Bouldin Index
CHS	Calinski–Harabasz Score
